# Nomogram integrating gene expression signatures with clinicopathological features to predict survival in operable NSCLC: a pooled analysis of 2164 patients

**DOI:** 10.1186/s13046-016-0477-x

**Published:** 2017-01-05

**Authors:** Jian Wu, Lizhi Zhou, Lixia Huang, Jincui Gu, Shaoli Li, Baomo Liu, Jinlun Feng, Yanbin Zhou

**Affiliations:** 1 Department of Respiratory Medicine, The First Affiliated Hospital, Sun Yat-sen University, No.58 Zhongshan Road 2, Guangzhou, Guangdong 510080 China; 2 Department of Biostatistics, School of Public Health, Southern Medical University, Guangzhou, China

**Keywords:** NSCLC, Gene expression signatures, NTP, CIBERSORT, Prognosis

## Abstract

**Background:**

The current tumor-node-metastasis (TNM) staging system is insufficient to predict outcome of patients with operable Non-Small Cell Lung Cancer (NSCLC) owing to its phenotypic and genomic heterogeneity. Integrating genomic signatures with clinicopathological factors may provide more detailed evaluation of prognosis.

**Methods:**

All 2164 clinically annotated NSCLC samples (1326 in the training set and 838 in the validation set) with corresponding microarray data from 17 cohorts were pooled to develop and validate a clinicopathologic-genomic nomogram based on Cox regression model. Two computational methods were applied to these samples to capture expression pattern of genomic signatures representing biological statuses. Model performance was measured by the concordance index (C-index) and calibration plot. Risk group stratification was proposed for the nomogram.

**Results:**

Multivariable analysis of the training set identified independent factors including age, TNM stage, combined prognostic classifier, non-overlapping signature, and the ratio of neutrophil to plasma cells. The C-index of the nomogram for predicting survival was statistically superior to that of the TNM stage (training set, 0.686 vs 0.627, respectively; *P* < .001; validation set, 0.689 vs 0.638, respectively; *P* < .001). The calibration plots showed that the predicted 1-, 3- and 5-year survival probabilities agreed well with the actual observations. Stratifying patients into three risk groups detected significant differences among survival curves.

**Conclusions:**

These findings offer preliminary evidence that genomic data provide independent and complementary prognostic information and incorporation of this information can refine prognosis in NSCLC. Prospective studies are required to further explore the value of this composite model for prognostic stratification and tailored therapeutic strategies.

**Electronic supplementary material:**

The online version of this article (doi:10.1186/s13046-016-0477-x) contains supplementary material, which is available to authorized users.

## Background

Lung cancer is a major cause of morbidity and mortality worldwide, with non-small cell lung cancer (NSCLC) accounting for around 4 in 5 new diagnoses [1]. Treatment decisions and prognosis for patients with NSCLC are largely driven by the assessment of the tumor-node-metastasis (TNM) staging system [2]. Currently, stage IA patients do not receive postoperative chemotherapy due to no survival advantage and its potential therapeutic toxicity [2, 3]. With respect to stage IB patients, disagreement prevails regarding which patients might be appropriate candidates for adjuvant therapy [3–5]. Although radical resection and adjuvant treatment extended cancer-specific survival of patients with stage II or IIIA disease, long-term prognosis continues to be jeopardized by the high risk of subsequent recurrence and drug toxicity [3, 5, 6]. Therefore, it remains a largely unmet need to reduce 30–50% rate of recurrence [7] to improve survival of these patients. Moreover, the identification of patients at high risk of recurrence and death, who are most likely to benefit from aggressive systemic therapy, is absolutely critical.

Rational application of adjuvant therapy requires accurate prognosis prediction for patients with resectable NSCLC. Numerous clinicopathological schemes have been proposed to optimize the staging system by introducing other prognostic variables, such as age, gender, and tumor lymphocytic infiltration [8–10]. Nonetheless, such models may have hit the ceiling and they still lack genomic information, which can add prognostic value to clinical models in lung cancer [11]. Recently, several gene expression signatures have been used to predict clinical outcomes in human cancer [12–19]. These genomic signatures may reflect specific biological/microenvironmental features of this underlying heterogeneous disease that can determine the tumor phenotype. However, these signatures often focus on one or several distinct aspects of tumor heterogeneity and few studies have attempted to demonstrate the power of combining all related clinicopathological factors with biological/microenvironmental features, which may render more precise information concerning risk assessment [20].

However, a simple, user-friendly and reliable nomogram incorporating genomic information with the traditional risk factors, widely validated in different cohorts, is needed to refine the prognosis of postoperative patients with stage I-IIIA NSCLC. Here, We aimed the following: (1) to build a composite clinicopathologic-genomic nomogram by systematically analyzing factors with potential prognostic value in a pooled cohort of 1326 postoperative NSCLC patients; (2) to assess the added value of genomic information compared with standard TNM staging, clinicopathological model and genomic model; and (3) to externally validate this nomogram by another independent pooled cohort. Finally, we constructed a composite model which can robustly identify patients at high risk of death and it was shown to outperform those based on clinicopathological variables or gene signatures alone.

## Methods

### Patients and samples

To identify gene expression data arrayed using Affymetrix Human Genome U133A or U133A plus 2.0 with clinically annotated data, we systematically searched Gene Expression Omnibus (GEO), The Cancer Genome Atlas (TCGA), ArrayExpress, caArray and related literature with the terms “lung cancer”, “NSCLC”, “lung adenocarcinoma”, “lung squamous cell carcinoma”, “survival”, “relapse”, “recurrence”, “prognostic” and “prognosis”. For some datasets whose clinical data were not with their gene expression profiles, we either searched the supplements or contacted one or more of the investigators to get the missing information. Raw microarray data and corresponding clinical data of these datasets were retrieved and manually organized when available. Only patients diagnosed with stage I-IIIA NSCLC, and with clinicopathological and survival information available, were included. We excluded patients who had follow-up time or survival time less than 1 month. In addition, patients with any missing or insufficient data on age, gender or histology were also excluded from subsequent processing. All of those studies previously were approved by their respective institutional review boards. Our study followed the Reporting Recommendations for Tumor Marker Prognostic Studies (REMARK) criteria as listed in their guidelines [21] and guidelines for prognostic factor studies in NSCLC [11].

### Data processing

Where raw CEL files from Affymetrix Human Genome U133A/Plus2 were available, data were normalized and annotated using an MAS5 algorithm and corresponding annotation files from R Bioconductor to obtain summarized values for each probeset, and otherwise we used pre-processed data as provided by the contributors. For each sample in all data sets, measurements without a gene annotation were excluded and multiple probesets corresponding a single gene were summarized into a gene symbol by taking the most variable probeset measured by interquartile range (IQR).

### Identification of genomic signatures with potential to predict prognosis

We queried literature database to identify gene expression signatures in studies where the prognostic value in human cancer or lung cancer were reported. The probesets or genes of those signatures were re-annotated using SOURCE web tool (http://source-search.princeton.edu/cgi-bin/source/sourceBatchSearch) to deal with the retired gene symbols and their differences in tested platforms.

### Statistical analysis

#### Subclass prediction

The preprocessed gene data were classified with gene expression signatures identified above using the Nearest Template Prediction (NTP) method as implemented in the Gene Pattern software (Broad Institute of Harvard and MIT, Boston, MA) [22]. NTP required only a list of pre-specified template signature genes and a dataset to be tested, without requiring corresponding training dataset, to capture the presence or absence of gene expression patterns in each sample. Briefly, a template of representative expression pattern of the signature genes was defined based on published gene signature from their respective study. Proximity of the signature genes expression pattern of the sample to the template was evaluated by calculating cosine distance. Significance of the proximity was assessed by a nominal p-value estimated based on a null distribution for the distance generated by randomly resampling the same number of signature genes from the sample genes 1000 times. False discovery rate (FDR) was used to correct the p-values for multiple hypothesis testing. The prediction analysis was performed separately for each dataset. A prediction of presence or absence of related signature was determined based on prediction FDR <0.05, and the rest of the samples with an intermediate expression level of the correlated genes in the signature were classified as uncertainty. Concordance among these predictions was evaluated using unsupervised clustering according to Cramer’s V coefficient of the paired prediction overlap as previously described [23]. Cramer’s V statistic values range from 0 to 1, values between 0.36 and 0.49 indicating substantially correlated and values higher than 0.5 strongly correlated.

### Enumeration of hematopoietic cells subsets from gene expression profiles

To quantify the relative abundances of 22 tumor-associated Leukocyte (TAL) subsets, we employed Cell type Identification By Estimating Relative Subsets Of known RNA Transcripts (CIBERSORT) method (500 iterations) and the LM22 gene signature which allowed for highly sensitive and specific discrimination of hematopoietic cells and were well-designed and validated on gene expression profiles from Affymetrix Human Genome U133A/Plus2 [24]. Subsequently, we aggregated 22 leukocyte subsets into 11 immune populations for clarity. The proportions of immune cells were predicted in each dataset separately.

### Development, comparison, and validation of prognostic models

The samples were separated into training/validation sets based on cohorts for identifying and evaluating the predictors. Overall survival (OS) was calculated from the date of diagnosis or surgery to the date of death or last follow-up. Patients who were alive at 5 years were administratively censored with OS as 5 years. Continuous variables were expressed as median (IQR) or median (range), and group comparison was performed by the t-test or the Wilcoxon rank sum test. Categorical variables were expressed as percentages, and group comparison was performed by Pearson’s χ^2^ test or the Fisher’s exact test. Median follow-up was calculated using the reverse Kaplan-Meier method [25]. Clinical variables previously shown of prognostic value (age, gender, histologic subtype and TNM stage) and all genomic factors identified with P values less than 0.05 in the univariate log-rank test were separately entered into a clinical Cox model and a genomic Cox model to compare the prognostic efficacy of clinical and genomic strategies. Clinicopathological and genomic factors identified above were then introduced into the multivariable analyses via the backward stepwise Cox proportional hazard model, from which coefficients were used to develop the composite nomogram. The whole population was divided into three risk groups (high, intermediate, low) according to the tertiles of the total scores given by the established nomogram in the training set. The discrimination and calibration of the nomogram for 1-, 3-, and 5-year were measured by the concordance index (C-index) and by calibration plot comparing the expected and observed survival probabilities respectively. For internal validation, the bootstrapping technique was used to adjust for over-fitting and over-optimistic model performance. An optimism-corrected C-index using 1000 bootstrap samples created with replacement was reported. The bootstrap resampling procedure was detailed in Additional file 1. Finally, for external validation, the total scores of each patient of the independent validation set were calculated according to the proposed nomogram to verify its generalization. Three risk groups were determined by the tertiles defined in the training set, and the respective Kaplan-Meier survival curves were delineated. All statistical tests performed were two-sided and the *P* values less than 0.05 were considered as statistical significance. Data analyses were implemented using the GenePattern software (http://genepattern.broadinstitute.org/gp/), the CIBERSORT tool (http://cibersort.stanford.edu/) and the R statistical package (http://www.r-project.org/).

## Results

### Identification of eligible samples and gene signatures

**Fig. 1:**
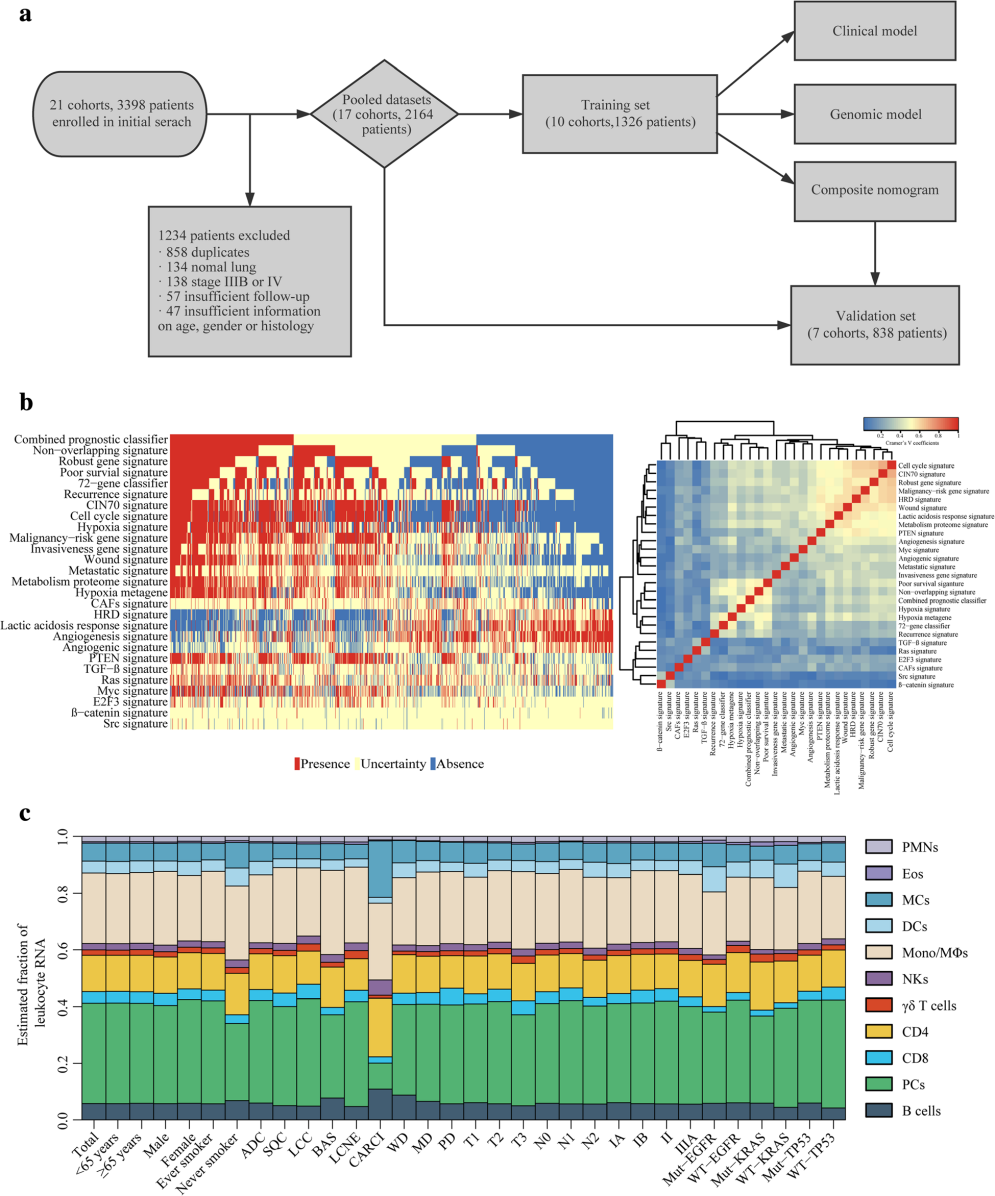
Genomic landscape of operable NSCLC based on gene expression profiling. **a** Flow chart of the study design. **b** Concordance of signature-based prediction result in the training set. Left panel: Each column represents the prediction of each individual sample. *Red*, *blue* and *pale yellow bars* indicate presence, absence and uncertainty prediction of the corresponding signature, respectively. Right panel: Heatmap of Cramer’s V coefficient showing correlation between these published signatures. **c** Consistency of estimated mRNA fractions of 11 tumor-associated Leukocyte (TAL) subsets, as calculated by CIBERSORT, within and across clinical subgroups of NSCLC in the training set

Predefined prognosis analysis was constrained to patients with early stages operable lung cancer. Of the 3398 patients from 21 previously established cohorts retrieved by the initial systematic search, 1234 were excluded because of duplicates, normal lung samples, or missing clinical information; the remaining 2164 clinically annotated NSCLC samples pertaining to 17 cohorts were considered eligible for inclusion in this study (Fig. 1a). All of the eligible patients were then divided into training/validation sets in terms of cohorts to develop and externally validate the composite model. Ultimately, a total of 1326 samples from ten cohorts were assigned to a training set to develop the model, and 838 samples from other seven cohorts to an independent validation set. Information about the datasets entering for our analysis and relevant clinicopathological characteristics of the patients included in the training set and validation set were shown in Additional file 2 and Table 1.

**Table 1:** Characteristics of patients and tumors included in the study

Characteristic	Training set (*N* = 1326)		Validation set (*N* = 838)		*P* value
Age (years)					
Median (IQR)	64	(57, 70)	65	(58, 71)	.003*
<65	697	(52.6)	403	(48.1)	.047**
≥65	629	(47.4)	435	(51.9)	
Gender					
Male	801	(60.4)	511	(61.0)	.826**
Female	525	(39.6)	327	(39.0)	
Smoking					
Ever smoker	907	(68.4)	578	(69.0)	.001**
Never smoker	177	(13.3)	148	(17.7)	
Unknown	242	(18.3)	112	(13.4)	
Histology					
Adenocarcinoma	880	(66.4)	420	(50.1)	< .001**
Squamous cell carcinoma	299	(22.5)	379	(45.2)	
Large cell carcinoma	54	(4.1)	7	(0.8)	
Basaloid tumors	31	(2.3)	32	(3.8)	
Large cell neuroendocrine carcinoma	41	(3.1)			
Carcinoid Tumors	21	(1.6)			
Grade					
Well differentiated	289	(21.8)	69	(8.2)	< .001**
Moderately differentiated	192	(14.5)	68	(8.1)	
Poorly differentiated	85	(6.4)	33	(3.9)	
Unknown	760	(57.3)	668	(79.7)	
Tumor stage					
T1	456	(34.4)	319	(38.1)	< .001**
T2	722	(54.4)	386	(46.1)	
T3	74	(5.6)	39	(4.7)	
Unknown	74	(5.6)	94	(11.2)	
Nodal status					
N0	908	(68.5)	583	(69.6)	< .001**
N1	239	(18.0)	135	(16.1)	
N2	102	(7.7)	26	(3.1)	
Unknown	77	(5.8)	94	(11.2)	
TNM stage					
IA	373	(28.1)	287	(34.2)	< .001**
IB	506	(38.2)	279	(33.3)	
II	290	(21.9)	220	(26.3)	
IIIA	157	(11.8)	52	(6.2)	
EGFR					
Mutant type	54	(4.1)	127	(15.2)	< .001**
Wild type	58	(4.4)	99	(11.8)	
Unknown	1214	(91.6)	612	(73.0)	
KRAS					
Mutant type	15	(1.1)	30	(3.6)	< .001**
Wild type	67	(5.1)	263	(31.4)	
Unknown	1244	(93.8)	545	(65.0)	
ALK fusion					
Yes	0		11	(1.3)	NA
No	0		215	(25.7)	
Unknown	1326	(100)	612	(73.0)	
TP53					
Mutant type	116	(8.7)	54	(6.4)	< .001**
Wild type	62	(4.7)	10	(1.2)	
Unknown	1148	(86.6)	774	(92.4)	
Median (range) follow-up (months)	73.4	(1, 256)	61.9	(1, 252)	< .001*
Status					
Alive	757	(57.1)	564	(67.3)	< .001**
Dead	569	(42.9)	274	(32.7)	

Data are number (%), unless otherwise indicated. *NA* not available. * *P* value for difference between medians; Wilcoxon rank sum test. ** *P* value for difference between categories; Pearson’s χ^2^ test

In the training set of 1326 patients with stage I-IIIA NSCLC, the majority were male (801 [60.4%]) and smoker (907 [68.4%]), with a median age of 64 years (IQR 57–70). Most patients had stage I disease (879 [66.3%]) and adenocarcinoma (880 [66.4%]) was the predominant histology. During a median follow-up times of 73.4 months (range 1–256), there were 569 deaths within the first 5 years, corresponding to 42.9% of the enrolled patients. Unlike the patients in the training set, patients in the validation set were older (median age was 65 years [IQR 58–71]; *P* = .003) and had a lower proportion of adenocarcinoma (420 [50.1%]) and deaths within the first 5 years (274 [32.7%]; *P* < .001). Median follow-up time for patients in the validation set was 61.9 months (range 1–252; *P* < .001).

Gene expression signatures might reflect diverse pathways, biological processes or other functions related to the heterogeneous tumor biology and microenvironment [26]. As different signatures may be active in different individual tumors, expression analyses of gene clusters could prove more revealing than single gene analyses [27]. Accordingly, all 27 gene expression signatures with potential prognostic value in lung cancer were identified from literature review, with the genes being clearly described in their respective studies [12–19, 28–42] (Table 2). Based on its initial purpose, we then classified these gene signatures into three major functional groups: (1) prognosis related signatures: six gene sets for predicting prognosis of lung cancer [28–33]; (2) biology/microenvironment related signatures: 14 gene clusters reflecting tumor biology or microenvironment state [12–17, 34–41]; (3) pathway related signatures: seven previously reported gene expression signatures to probe the status of oncogenic pathways [18, 19, 42]. Additionally, an association between cancer survival and abundance of diverse TAL subsets had been recently proposed by applying a computational approach known as CIBERSORT, which can use gene expression data to enumerate and characterize infiltrating immune cells in bulk tumor [43]. For example, tumor-associated neutrophil and plasma cell signatures exhibited opposite prognostic associations in lung cancer. We reasoned that these signatures would allow the description of tumor heterogeneity [43].

**Table 2:** Genomic Signatures Included in the Study

Functional class	Signature name	Year	Number of genes mapped onto the platforms		Patients with the signature classified as (%)^a^			Reference
			GPL570	GPL96	Presence	Uncertainty	Absence	
Prognosis	Combined prognostic classifier	2006	91	90	370 (27.9)	547 (41.3)	409 (30.8)	[28]
Prognosis	Non-overlapping signature	2008	86	85	393 (29.6)	532 (40.1)	401 (30.2)	[29]
Prognosis	Robust gene signature	2011	58	57	467 (35.2)	358 (27.0)	501 (37.8)	[30]
Prognosis	Poor survival signature	2006	61	60	330 (24.9)	577 (43.5)	419 (31.6)	[31]
Prognosis	72-gene classifier	2009	59	47	429 (32.4)	437 (33.0)	460 (34.7)	[32]
Prognosis	Recurrence signature	2011	43	43	253 (19.1)	814 (61.4)	259 (19.5)	[33]
Biology/microenvironment	CIN70 signature	2006	70	66	609 (45.9)	151 (11.4)	566 (42.7)	[12]
Biology/microenvironment	Cell cycle signature	2002	729	601	599 (45.2)	112 (8.4)	615 (46.4)	[34]
Biology/microenvironment	Hypoxia signature	2006	141	117	504 (38.0)	323 (24.4)	499 (37.6)	[13]
Biology/microenvironment	Malignancy-risk signature	2011	91	91	547 (41.3)	644 (48.6)	135 (10.2)	[35]
Biology/microenvironment	Invasiveness gene signature	2007	172	128	315 (23.8)	798 (60.2)	213 (16.1)	[36]
Biology/microenvironment	Wound signature	2004	411	348	459 (34.6)	486 (36.7)	381 (28.7)	[14]
Biology/microenvironment	Metastatic signature	2002	115	112	214 (16.1)	1042 (78.6)	70 (5.3)	[15]
Biology/microenvironment	Metabolism proteome signature	2014	194	187	412 (31.1)	586 (44.2)	328 (24.7)	[37]
Biology/microenvironment	Hypoxia metagene	2007	142	123	378 (28.5)	538 (40.6)	410 (30.9)	[38]
Biology/microenvironment	CAFs signature	2011	45	34	176 (13.3)	1074 (81.0)	76 (5.7)	[39]
Biology/microenvironment	HRD signature	2014	217	182	208 (15.7)	590 (44.5)	528 (39.8)	[16]
Biology/microenvironment	Lactic acidosis response signature	2008	1770	1378	415 (31.3)	463 (34.9)	448 (33.8)	[17]
Biology/microenvironment	Angiogenesis signature	2013	477	399	505 (38.1)	573 (43.2)	248 (18.7)	[40]
Biology/microenvironment	Angiogenic signature	2005	58	56	215 (16.2)	928 (70.0)	183 (13.8)	[41]
Pathway	PTEN signature	2007	177	156	459 (34.6)	688 (51.9)	179 (13.5)	[18]
Pathway	TGF-β signature	2008	240	212	161 (12.1)	1042 (78.6)	123 (9.3)	[19]
Pathway	Ras signature	2006	252	203	321 (24.2)	869 (65.5)	136 (10.3)	[42]
Pathway	Myc signature	2006	194	159	348 (26.2)	712 (53.7)	266 (20.1)	[42]
Pathway	E2F3 signature	2006	241	183	149 (11.2)	1129 (85.1)	48 (3.6)	[42]
Pathway	β-catenin signature	2006	76	70	8 (0.6)	1291 (97.4)	27 (2.0)	[42]
Pathway	Src signature	2006	63	57	17 (1.3)	1280 (96.5)	29 (2.2)	[42]

^a^Samples were classified as presence, absence or uncertainty by respective published genomic signatures based on prediction result (false discover rate [FDR] <0.05) of Nearest Template Prediction (NTP)

### Genomic landscape of early stage NSCLC

Among the 27 signatures evaluated, all of them were able to confidently classify patients (FDR <0.05) into their predicted presence or absence subclass. Table 2 and Fig. 1b summarizes the prediction result obtained for each of the 1326 patients. The cell cycle signature [34] was the most prevalent prediction in the training set (88.6%; 1214 of 1326), whereas the Src signature [42] was identified in only 3.5% (46 of 1326) of tumor samples. Interestingly, 290 of 1326 (21.9%) patients concomitantly harbored 5 or more presence/absence signatures. We then sought to evaluate the concordance of these 27 signatures using Cramer’s V coefficient. Signature unsupervised clustering based on these coefficients indicated a substantial association among the three predefined groups of signatures (Fig. 1b) as follows: (1) signatures reflecting biological and microenvironmental characteristics related to cell cycle, chromosomal instability, proliferation, homologous recombination defect, wound healing, acidosis, and metabolism, such as cell cycle signature [34], CIN70 signature [12], malignancy-risk signature [35], HRD signature [16], wound signature [14], lactic acidosis response signature [17], and metabolism proteome signature [37]; (2) signatures predicting lung cancer prognosis, such as combined prognostic classifier [28], non-overlapping signature [29], poor survival signature [31] and recurrence signature [33]; (3) signatures indicating patterns of specific pathway deregulation, e.g., TGF-β signature [19], RAS signature [42] and E2F3 signature [42]. The observation that some of them did not have a strong correlation implied that these signatures could capture complementary biological features essential in prognosis prediction.

The CIBERSORT method was applied to samples in the training set to quantify the relative proportions of 11 TAL subsets. As shown in Fig. 1c, CIBERSORT revealed striking consistency in relative immune cell fractions within and across clinical subgroups of lung cancer, with plasma cells, monocytes and macrophages, CD4 T cells, and mast cells being the most common immune cell subsets with mean fractions of 0.354, 0.249, 0.128 and 0.064 respectively. Patient samples were divided into 3 groups (low, medium, and high) according to the tertiles values of neutrophil-to-plasma cells fractions inferred in the training set, which were then applied to the validation set.

### Independent prognostic factors for survival in the training set

We then sought to test which factors were statistically significant for lung cancer-related death. The results of the univariate log-rank test are displayed in Additional file 3. Younger age (*P* < .001) and female gender (*P* = .012) were associated with favorable prognosis. Compared with the stage IA group, other groups (stage IB, stage II, and stage IIIA) were associated with poor prognosis, with a hazard ratio (HR) of 1.55 (95% CI 1.22–1.97; *P* < .001), 2.39 (95% CI 1.86–3.07; *P* < .001), and 4.21 (95% CI 3.22–5.50; *P* < .001), respectively. Oddly, there was no significant survival difference between adenocarcinoma and squamous cell carcinoma (*P* = .312). With respect to genomic variables, most of them were identified as significantly associated with a less-favorable prognosis, whereas the presence of HRD signature [16] (*P* < .001), lactic acidosis response signature [17] (*P* < .001), angiogenesis signature [40] (*P* < .001) and angiogenic signature [41] (*P* < .001) were associated with an improved survival. Of note, in concordance with the results previously reported [43], we observed a strong association between inferior survival and a higher ration of neutrophil to plasma cells (*P* < .001) in NSCLC.

**Fig. 2:**
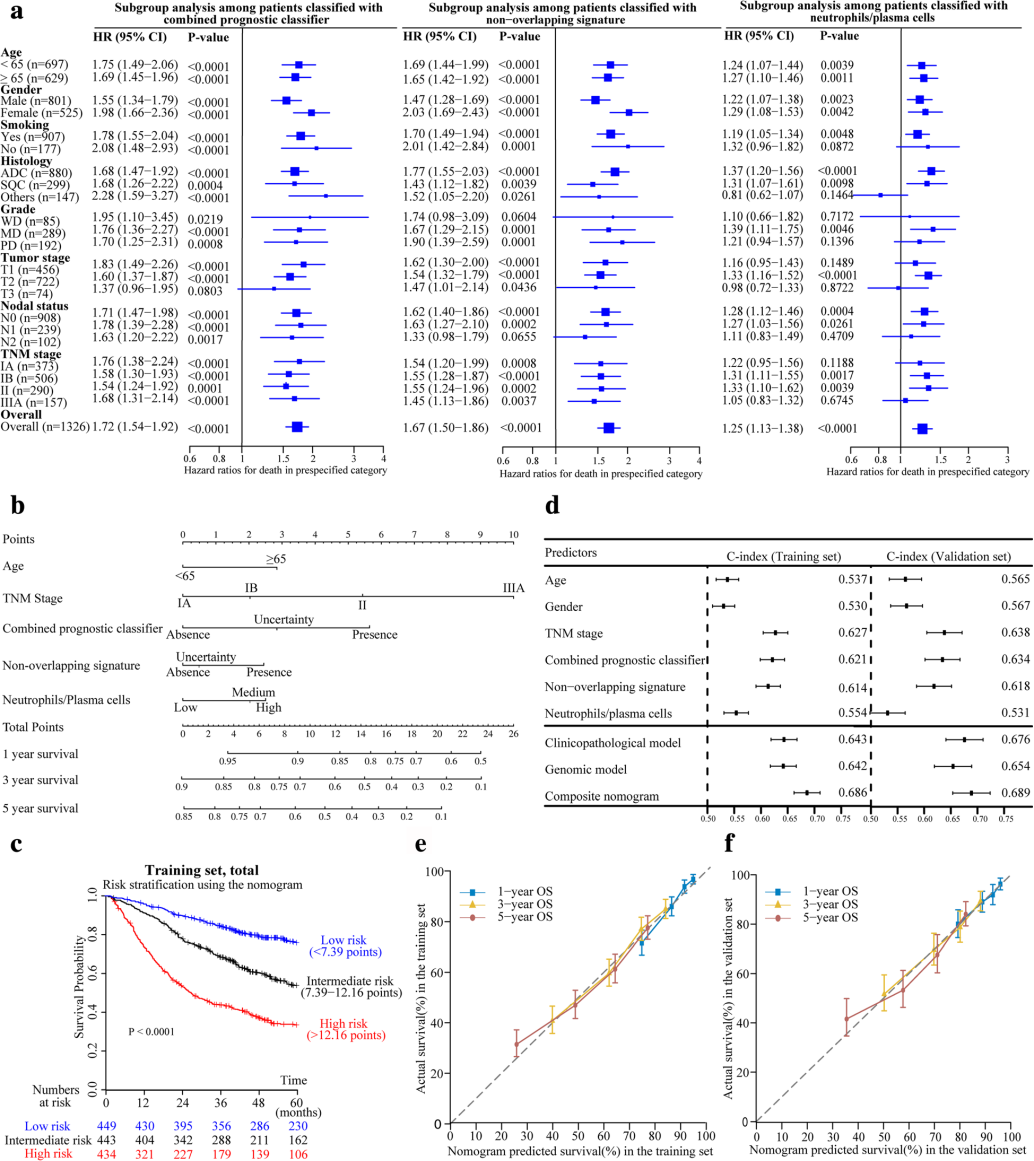
Development and validation of the composite clinicopathologic-genomic nomogram. **a** Subgroup analysis using the three genomic factors in the training set. Data were expressed using 5-year overall mortality hazard ratio (HR ± 95% confidence interval) for each stepwise increase in the level of predicted signature subclass (that is, absence to uncertainty and uncertainty to presence, or low to medium and medium to high). Square sizes are proportional to subgroup sizes. **b** Composite nomogram to predict survival for patients with operable NSCLC. **c** Kaplan-Meier survival curves of overall survival among risk stratification groups using the proposed nomogram in the training set. **d** Performance of models and individual variables as assessed by concordance index (C-index) in the training set and validation set for predicting postoperative survival for patients with NSCLC. **e**, **f** The calibration curves of the proposed nomogram for predicting overall survival (OS) at 1-, 3-, and 5-year in the training set (**e**) and in the validation set (**f**). *Squares* and *whiskers* represent individual data points and associated 95% confidence intervals, respectively

To assess the hypothesis that survival prediction models integrating gene signatures can better identify patients at high risk of death than those based on clinicopathological variables or genomic information alone, we developed a clinicopathological model, a genomic model and a composite model based on the training set using all significant factors in the univariable analysis (Table 3). Clinicopathological multivariable analyse demonstrated that age (*P* < .001), gender (*P* = .010) and TNM stage (*P* < .001) were independent risk factors for overall survival. By contrast, combined prognostic classifier [28] (*P* < .001), non-overlapping signature [29] (*P* = .001) and the ratio of neutrophil to plasma cells [43] (*P* = .012) were identified as independent predictors in the genomic model. We next sought to test the independent predictive value of the three genomic factors when confronted with known clinicopathological variables in the training set. Intriguingly, these genomic factors were significantly associated with overall survival in most of the subgroups analyzed, regardless of the age, gender, smoking history, grade, histology, tumor stage or lymph nodal status (Fig. 2a). Moreover, in patients categorized by the TNM stage, these genomic variables were also able to classify them into good and poor survival outcome especially in stage IA or stage IB. Furthermore, 2 out of 3 genomic factors were also prognostically significant within subsets of patients harboring either wild-type or activating mutation of EGFR, ALK, KRAS, or TP53 in the overall pooled datasets (see Additional file 4). Importantly, when age, gender, TNM stage, combined prognostic classifier [28], non-overlapping signature [29] and the ratio of neutrophil to plasma cells [43] were entered as covariates in a joint Cox model, combined prognostic classifier [28] (*P* < .001), non-overlapping signature [29] (*P* = .036) and the ratio of neutrophil to plasma cells [43] (*P* = .004) remained statistically significant in addition to age (*P* < .001) and TNM stage (*P* < .001) in the training set.

**Table 3:** Multivariate Cox regression models for overall survival in the training set^a^

Variable	Clinical multivariate analysis		Genomic multivariate analysis		Composite multivariate analysis	
	HR (95% CI)	*P* value	HR (95% CI)	*P* value	HR (95% CI)	*P* value
Age		<0.001				<0.001
<65	Reference				Reference	
≥65	1.41 (1.19–1.66)	<0.001			1.45 (1.23–1.71)	<0.001
Gender		0.010				
Female	Reference					
Male	1.25 (1.05–1.49)	0.010				
TNM6th stage		<0.001				<0.001
IA	Reference				Reference	
IB	1.48 (1.17–1.88)	0.001			1.30 (1.02–1.66)	0.031
II	2.34 (1.82–3.01)	<0.001			2.04 (1.58–2.63)	<0.001
IIIA	4.30 (3.29–5.62)	<0.001			3.70 (2.82–4.86)	<0.001
Combined prognostic classifier				<0.001		<0.001
Absence			Reference		Reference	
Uncertainty			1.38 (1.06–1.79)	0.016	1.45 (1.12–1.89)	0.006
Presence			1.98 (1.46–2.70)	<0.001	2.09 (1.54–2.86)	<0.001
Non-overlapping signature				0.001		0.036
Absence			Reference		Reference	
Uncertainty			1.22 (0.94–1.58)	0.142	1.07 (0.82–1.39)	0.633
Presence			1.68 (1.24–2.27)	0.001	1.38 (1.01–1.87)	0.040
Neutrophils/plasma cells				0.012		0.004
Low			Reference		Reference	
Medium			1.27 (1.03–1.57)	0.027	1.30 (1.06–1.61)	0.014
High			1.34 (1.09–1.65)	0.005	1.39 (1.13–1.71)	0.002

^a^Hazard ratio (HR) estimated by Cox proportional hazards regression. All statistical tests were two-sided. *CI* confidence interval

### Construction, comparison and validation of the composite nomogram

To develop a composite prognostic model, we built a nomogram that integrated the significant factors identified above to predict survival of patients with operable NSCLC (Fig. 2b). The nomogram demonstrated that the TNM stage had the largest contribution to prognosis, followed by the combined prognostic classifier [28] and non-overlapping signature [29]. Age and the ratio of neutrophil to plasma cells [43] showed a moderate effect on survival rate. Each category within these variables was assigned a point on the top scale based on the coefficients from Cox regression. By summing all of the assigned points for the five variables and drawing a vertical line between Total Points and survival probability axis, we were easily able to obtain the estimated probability of 1-, 3- and 5-year survival. The risk score cutoff values were selected to group patients in terms of total points in the training set into roughly equal tertiles, accurately divided patients into low, intermediate, and high risk (Fig. 2c). Samples were divided into 3 subgroups according to the tertiles of the total number of points in the training set: patients at low risk (<7.39 points), intermediate risk (7.39–12.16 points), and high risk (>12.16 points) of death.

To assess the added value of genomic information, we sought to compare the performance of the proposed nomogram with standard TNM staging, clinicopathological model and genomic model by applying them to the training set and validation set. Expectedly, the composite nomogram had the highest C-index compared with single prognostic variables, clinical model and genomic model in both the training and validation set (Fig. 2d). This suggested that the joint clinicopathologic-genomic model could attain superior prognostic performance than either clinicopathological or genomic information alone.

**Fig. 3:**
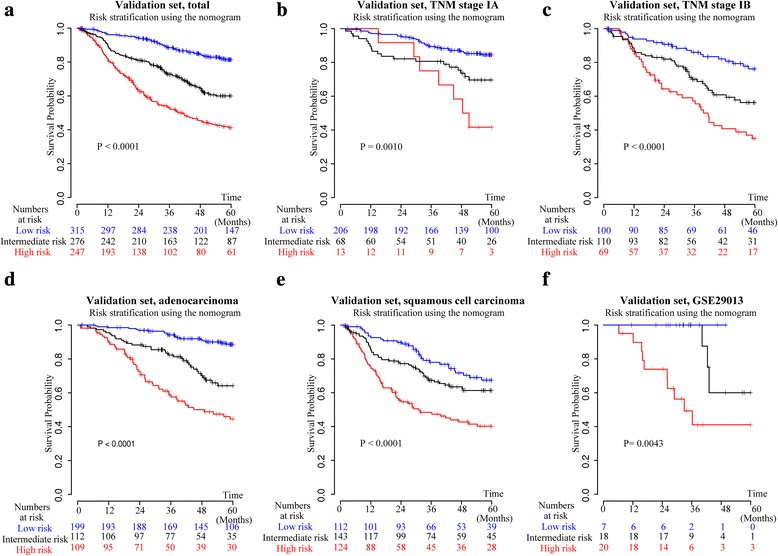
Kaplan-Meier survival curves of overall survival for patients in the validation set. **a**, all patients; **b**, stage IA; **c**, stage IB; **d**, adenocarcinoma; **e**, squamous cell carcinoma; **f**, GSE29013

Prognostic performance of the composite nomogram was further validated using bootstrap resampling and an independent validation set of 899 surgically resected NSCLC. In the training set, the C-index of this joint model (0.686, 95% CI 0.662–0.710) for predicting OS was statistically superior to that of TNM staging system (0.627, 95% CI 0.604–0.650; *P* < .001). Likewise, the C-index continued to be statistically higher for the nomogram (0.689, 95% CI 0.654–0.724) in the validation set than for the TNM stage (0.638, 95% CI 0.605–0.671; *P* < .001). The calibration plots showed that the predicted 1-, 3- and 5-year survival probabilities of the nomogram agreed well with the actual observations in both sets (Fig. 2e, f). Applying cutoff value from the training set can stratify patients in the validation set into three distinct risk subgroups with statistically significant difference in overall survival curves (Fig. 3a). Moreover, in patients categorized by major clinicopathological features, the survival rates predicted by the nomogram continued to illustrate significant distinctions between the Kaplan-Meier curves (Fig. 3b-f).

## Discussion

The genetic heterogeneity both between and within tumors poses significant challenges to predicting patient clinical outcome. Although the TNM staging system indicates the level of the malignant potential and disease progression and is a strong prognostic factor in NSCLC [9], but it has some shortcomings. Lung cancer patients with the same stage of disease can have remarkably different overall outcome following curative resection [8]. Moreover, the staging system strongly depends on the tumor size, the degree of lymph node involvement, and the extent of distant metastases, and it neither takes into account other clinicopathological risk factors, such as age, gender, or grade, nor does it considers the biological heterogeneity of NSCLC patients [2]. To address this, we pooled the largest clinically annotated NSCLC gene expression profiling datasets to date and separated it into training/validation sets to develop and validate a composite clinicopathologic-genomic nomogram for estimation of the risk of death in patients with early stage operable NSCLC. The proposed nomogram outperformed the prognostic potential of models based on clinicopathologic variables or gene signatures alone and could robustly stratify patients into three different prognosis groups with significantly different median survivals.

Each eligible cohort contained samples from a great majority of the resectable lung cancer patients who were diagnosed within a specific geographical region and time period and did not receive adjuvant therapy (see Additional file 2). The wide spatial and temporal distribution and relatively large sample size in this pooled cohorts enhanced its clinical and genomic representativeness and generalizability for NSCLC patients. However, it would be increasingly difficult to conduct a similar research project, since adjuvant therapies which would affect patient outcomes were recommended for most of the patients.

Despite evidence that clinicopathological factors (e.g. age, gender, histology) are prognostic relevant [8–10], the advent of high-throughput genomic profiling tools have enabled additional systematic evaluation of lung cancer genomic heterogeneity to aid prognosis [26, 27]. The comprehensiveness of such data provides an opportunity to dissect this heterogeneous and elusive disease entity into more homogeneous subgroups that can detect genomic phenotypes representing potential candidates for prognostic and predictive biomarkers, in ways that clinical risk factors cannot [26, 27]. Recent microarray studies have used gene expression signatures that reflect pathway activation, biological status or outcome prediction to predict clinical prognosis in human cancer and lung cancer [12–19, 28–42]. For instance, the malignancy-risk gene signature associated with both cancer risk and progression is composed of numerous cell cycle and DNA replication-related genes [35]. Aggressive tumors resulted in the development of an invasiveness gene signature that is correlated significantly with both overall survival and tumor recurrence [36]. Similarly, primary tumors harbored the metastases-related gene expression pattern are associated with poor outcome [15]. Furthermore, homologous recombination repair deficiency may contribute to cancer initiation and carcinogenesis [16]. Individuals with the HRD gene signature have significantly longer survival times compared with those without [16]. Differences in survival were found between lung cancer patients with and without DNA alterations in genes encoding the metabolism proteome [37]. Additionally, multiple oncogenic pathway statuses can stratify patients with different outcomes [42]. As for aspects of the tumor microenvironment, patients who have lung tumors that exhibit a gene expression pattern similar to that of serum-induced fibroblasts have a poor clinical outcome [14]. Hypoxia signature and lactic acidosis response signature are two strong prognostic factors for overall survival in multiple cancers [13, 17, 38]. Moreover, high expression of angiogenesis-related genes is associated with good prognosis in multiple cancer types [40, 41]. Finally, the prognostic potential of a leukocyte gene signature has been recently revealed by a computational approach known as CIBERSORT [43]. These genomic signatures which can help to refine the molecular classification and prognosis in human cancer include genes that exhibit coherently similar expression pattern, closely reflect specific biological processes, and reciprocally compensate each other in capturing the same patterning of biological dysregulation [22, 26, 27].

Indeed, the result of univariate analysis confirmed that most of the signatures were associated with survival prognosis, as stated in their respective publications (see Additional file 3). The observation that some of the patients concurrently harbored more than one poor-outcome signatures (Fig. 1b) indicated that their tumors were genetically unstable and compatible with an aggressive disease. This may partially explain wide nonoverlapping among the prognosis-related signatures identified in various lung cancer microarray studies [28–32]. Another explanation was these signatures might reflect similar biological behavior. Importantly, unsupervised clustering analysis of these signatures identified 3 subgroups: one subgroup defining biological and microenvironmental features, another subgroup predicting lung cancer prognosis, and a third indicating oncogenic pathway status (Fig. 1b). Despite the strong association between some signatures within these subgroups, the predicted result varied considerably owing to interpatient heterogeneity in the biological status of the samples. Multiple, different biological statuses were regularly present in the individual tumor within predicted outcome-related categories (Fig. 1b). For example, samples predicted as good outcome in prognosis-related signatures [28–33] exhibited a gene expression pattern similar to HRD signature [16] and angiogenesis signature [40]. It was in high concordance with previous reports correlating HRD signature [16] and angiogenesis signature [40] with good outcome in lung cancers. In contrast, an increased invasive [36], proliferative [34, 35], metastatic [15] and hypoxic [13, 38] response was present in samples predicted as poor outcome in prognosis-related signatures [28–33]. To sum up, these genomic signatures reflecting multiple distinct biological aspects of tumor heterogeneity may dissect the complexity and interplay between cancer cells and stromal cells, capture complementary biological states, and thus help to refine prognosis in early stage NSCLC. Additionally, identification of prognostically distinct subgroup of patients with specifically featured candidates using genomic profiling-based signatures may provide an opportunity for selection of therapeutic strategies tailored to the individual patterns of the biological state (e.g. oncogenic pathway activation, hypoxia, wound, and metabolism) [42].

However, our primary goal was to unravel the prognostic power of these reported genomic signatures coupled with clinicopathological variables in a large cohort of early stage NSCLC. Multivariable analysis in the training set identified age, TNM stage, combined prognostic classifier, non-overlapping signature, and the ratio of neutrophil to plasma cells as independent prognostic factors (Table 3), which were highly consistent with studies concerning risk factors in lung cancer [8, 9, 28, 29, 43]. The combined prognostic classifier was enriched for genes reflecting epidermal differentiation, signaling, cell cycle and growth, transcription, translation and metabolism [28]; contrastingly, non-overlapping signature contained genes involved in cell movement, cell death, cell cycle, and signaling processes [29], supporting that it captures complementary prognostic information in NSCLC. Subsequent subgroup analysis confirmed the independent prognostic value when confronted with known clinicopathological variables (Fig. 2a). Accordingly, we cautiously decided to use the five significant prognostic factors (age, TNM stage, combined prognostic classifier [28], non-overlapping signature [29], and the ratio of neutrophil to plasma cells [43] to build the composite nomogram (Fig. 2b). For validation of this nomogram to prevent overfitting and to verify generalization, the C-index and calibration plots were used. The C-index of the nomogram demonstrated a superior prognostic value over the sixth AJCC TNM classification in the training set and the validation set (Fig. 2c). The calibration plots showed optimal agreement between the expected and observed survival probabilities in both sets (Fig. 2e, f), insuring the reliability and repeatability of the proposed nomogram. In addition, three risk groups were defined on the basis of total points by using tertiles in the training set (Fig. 2d). Using these tertiles, we stratified patients from diverse geographical and ethnic origins into three distinct risk subgroups (Fig. 3). A prognostic model for NSCLC can be considered clinically useful if it is either 1) more effective than standard prognostic factors in identifying high risk subgroup of stage I patients who might benefit from adjuvant chemotherapy or 2) identifies low risk stage II patients who may spare needless drug toxicity [11]. The proposed nomogram can identify those patients that are most likely to be misclassified by clinical models, especially for patients with stage IB disease to whom the role of chemotherapy currently remains controversial (Fig. 3c). Of note, the nomogram could also be used in the cohort of GSE29013 which was based on analysis of FFPE specimens [30], indicating its clinical application in a broad and practical tissue source (Fig. 3f). Different treatment and follow-up strategies may be appropriate for each of the three categories. For example, more intense adjuvant therapy or new treatment regimen could be given to patients in the high risk group. Similarly, in the intermediate risk group, intensive follow-up or standard adjuvant therapy of these patients was clearly essential. Low risk patients were likely cured by surgery and might not be candidates for systemic therapy. Thus complications deriving from potentially unnecessary adjuvant therapy and life-threatening progression owing to insufficient treatment could both be avoided.

Different accrual times and technologies differences may introduce potential confounding effects. Therefore, Affymetrix Human Genome U133A and Affymetrix Human Genome U133 Plus2 are included only, because they have 22,277 probesets in common to measure the same gene with the same specificity, sensitivity, and dynamic range and because the sample size is sufficient to detect an important difference between the proposed nomogram and TNM staging system. Furthermore, this study did not overcome limitations related to patient comorbidities, treatment heterogeneity, retrospective nature with potential for error or bias, and unbalancement of clinicopathological features between the training set and the validation set. Also, we noted that prognosis of patients with NSCLC was better in the validation set than in the training set. A reason for this might be that the two sets had significant geographical and clinicopathologic differences in distribution. Especially, the training set included more stage IIIA patients (11.8% vs. 6.2%; Table 1). Another relevant issue was that some prognostic factors (e.g., gender [8], histology [8], and smoking status [44]) were not included in the model owing to not reaching statistical significance in the training set or insufficient data. Still, the forthcoming 8^th^ edition of the TNM classification of lung cancer [45] may perform better than either the current 7^th^ edition or the 6^th^ edition used in our study. Direct comparison of the different versions of the TNM staging is ideal for accuracy comparison. Unfortunately, we don’t have much data for direct comparison in our study. But, we do find some references about the accuracy comparison. A slight increase in the value for R^2^, which is an estimate of the percent variance explained by the Cox proportional hazards regression models, was observed for both the newly edition when compared to the previous one [45, 46]. However, in a Norwegian cancer registry series contained 1885 patients from 2001 to 2005, the c-index was 0.68 for both the current 7th edition and the previous edition, indicating no difference in their predictive accuracy [47]. These factors were important for survival for patients with early stage lung cancer and their incorporation may further improve the prognostic stratification of patients. Another potential shortcoming was that the panel of signatures representing biological status was not exhaustive and a more sensitive, specific and efficient prediction method to capture the presence of absence of these signatures was needed. Currently, patients with advanced NSCLC are generally treated with systemic therapy or a symptom-based palliative approach. Therapy for these patients should be guided by the mutation status of the tumor whenever possible. Therefore, this nomogram is not suitable for late stage patients.

## Conclusions

The improved performance of this combinatorial scheme accentuated the importance of integrating all aspects of the tumor biology and microenvironment into prognostic stratification. Pending further prospective clinical validation, these results provide preliminary evidence that the genomic information could be combined with clinical data to help refine patient prognosis and should be considered as an independent and complementary approach to the current clinicopathologic prognostic model. Ultimately, further studies on prospective data collection and inclusion of some other factors are required to validate and optimize this model.

## Additional files

Additional file 1: Bootstrap resampling procedure for internal validation (TIF 1594 kb)
Additional file 2: Characteristics of cohorts included in the study. (DOCX 37 kb)
Additional file 3: Univariable associations of clinicopathologic and genomic factors with risk of 5-year overall mortality in the training set. (XLSX 12 kb)
Additional file 4: Prognostic value of the three genomic factors within mutational subsets of NSCLC in the overall pooled dateses, training set and validation set. (XLSX 12 kb)

## Abbreviations

ADC: Adenocarcinoma
BAS: Basaloid tumors
CARCI: Carcinoid tumors
CIBERSORT: Cell type identification by estimating relative subsets of known RNA transcripts
C-index: Concordance index
DCs: Dendritic cells
Eos: Eosinophils
FDR: False discovery rate
LCC: Large cell carcinoma
LCNE: Large cell neuroendocrine carcinoma
MCs: Mast cells
MD: Moderately differentiated
Mono/MΦs: Monocytes and macrophages
Mut: Mutant
NKs: Natural killer cells
NSCLC: Non-small cell lung cancer
NTP: Nearest template prediction
OS: Overall survival
PCs: Plasma cells
PD: Poorly differentiated
PMNs: Neutrophils
SQC: Squamous cell carcinoma
TNM: Tumor-node-metastasis
WD: Well differentiated
WT: Wild type
