# Supplementary figures and images for: Nomogram integrating gene expression signatures with clinicopathological features to predict survival in operable NSCLC: a pooled analysis of 2164 patients
Source: J Exp Clin Cancer Res. 2017 Jan 5;36:4. doi: 10.1186/s13046-016-0477-x (PMC5216590; doi:10.1186/s13046-016-0477-x)

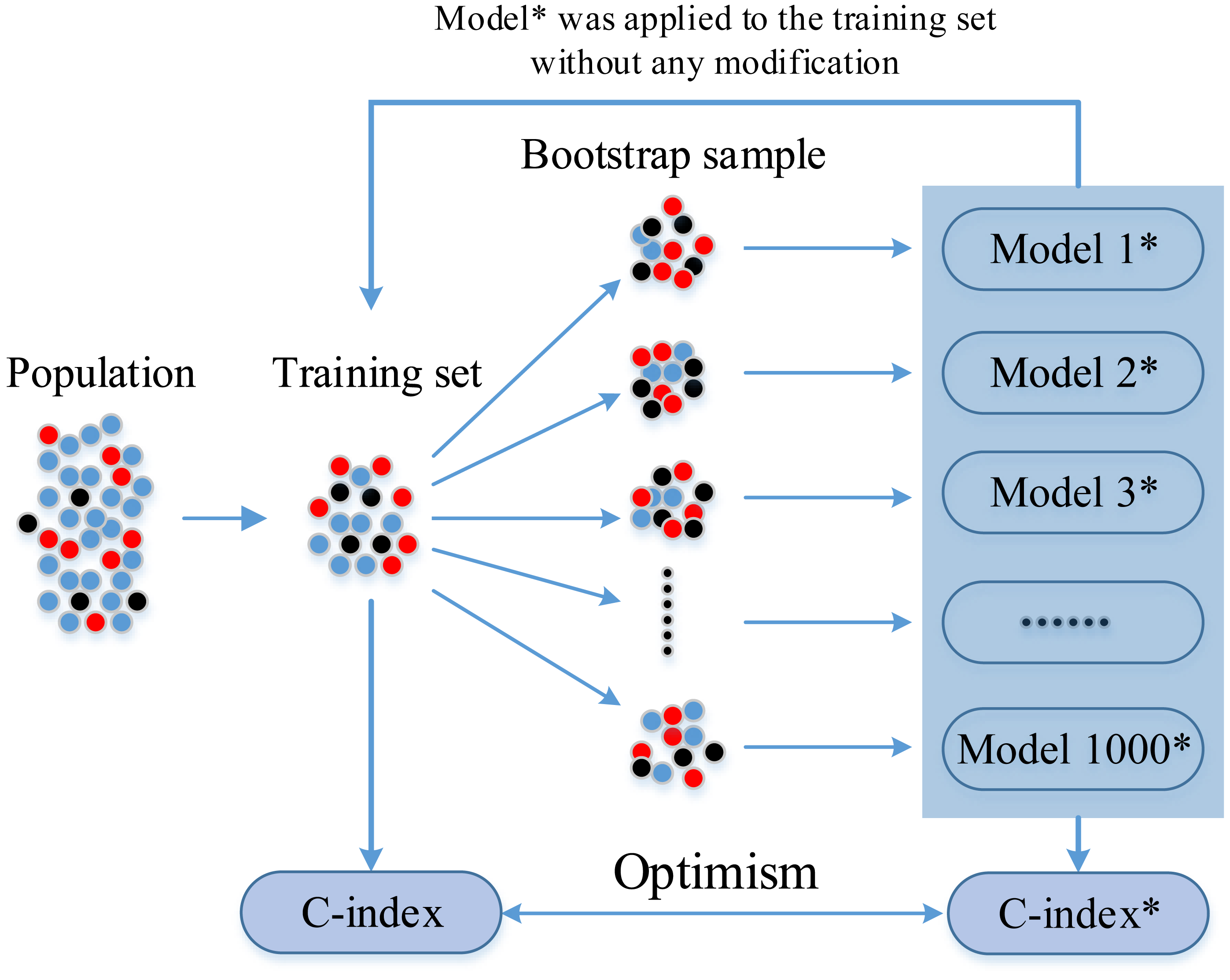

Supplement: Additional file 1: — Bootstrap resampling procedure for internal validation (TIF 1594 kb) [file 13046_2016_477_MOESM1_ESM.tif]
