# Supplementary material for: Nomogram integrating gene expression signatures with clinicopathological features to predict survival in operable NSCLC: a pooled analysis of 2164 patients
Source: J Exp Clin Cancer Res. 2017 Jan 5;36:4. doi: 10.1186/s13046-016-0477-x (PMC5216590; doi:10.1186/s13046-016-0477-x)
Supplement: Additional file 2: — Characteristics of cohorts included in the study. (DOCX 37 kb) [file 13046_2016_477_MOESM2_ESM.docx]

Supplement table 1: Characteristics of cohorts included in the study.

| Dataset | Repository | Country | Raw data  available | Platform | Accrual | No. of Patients  enrolled | Tissue type | Use in the present  study for | Reference |
| --- | --- | --- | --- | --- | --- | --- | --- | --- | --- |
| GSE8465/ca00182 | GEO/caArray | USA | Yes | GPL96 | 1996-2006 | 425 | Fresh/frozen | training | (1) |
| GSE30219 | GEO | France | Yes | GPL570 | - | 238 | Fresh/frozen | training | (2) |
| GSE37745 | GEO | Sweden | Yes | GPL570 | 1995-2005 | 184 | Fresh/frozen | training | (3) |
| GSE8894 | GEO | South Korea | No | GPL570 | 1995-2005 | 126 | Fresh/frozen | training | (4) |
| GSE14814 | GEO | Canada | Yes | GPL96 | 1994-2004 | 89 | Fresh/frozen | training | (5) |
| E-MTAB-923 | EBI | France | Yes | GPL570 | 2002-2008 | 84 | Fresh/frozen | training | (6) |
| GSE19188 | GEO | Netherlands | Yes | GPL570 | 1992-2004 | 76 | Fresh/frozen | training | (7) |
| GSE10245 | GEO | Germany | Yes | GPL570 | - | 57 | Fresh/frozen | training | (8) |
| GSE31908 | GEO | USA | Yes | GPL96 | 1991-2001 | 29 | Fresh/frozen | training | - |
| GSE6253 | GEO | USA | Yes | GPL96 | 1997-2001 | 18 | Fresh/frozen | training | (9) |
| GSE31210 | GEO | Japan | Yes | GPL570 | 1998-2008 | 226 | Fresh/frozen | validation | (10) |
| GSE50081 | GEO | Canada | Yes | GPL570 | 1996-2005 | 177 | Fresh/frozen | validation | (11) |
| GSE4573 | GEO | USA | No | GPL96 | 1991-2002 | 123 | Fresh/frozen | validation | (12) |
| TCGA/GSE68793 | TCGA/GEO | Multinational | Yes | GPL96 | - | 104 | Fresh/frozen | validation | - |
| GSE3141 | GEO | USA | No | GPL570 | - | 83 | Fresh/frozen | validation | (13) |
| E-MTAB-2435/ -1727 | EBI | France | Yes | GPL570 | 1988-2006 | 80 | Fresh/frozen | validation | (14) |
| GSE29013 | GEO | USA | Yes | GPL570 | 2001-2005 | 45 | FFPE | validation | (15) |

**Supplementary References**

1. Director's Challenge Consortium for the Molecular Classification of Lung A, Shedden K, Taylor JM, Enkemann SA, Tsao MS, Yeatman TJ, et al. Gene expression-based survival prediction in lung adenocarcinoma: a multi-site, blinded validation study. Nat Med. 2008;14:822-7.

2. Rousseaux S, Debernardi A, Jacquiau B, Vitte AL, Vesin A, Nagy-Mignotte H, et al. Ectopic activation of germline and placental genes identifies aggressive metastasis-prone lung cancers. Sci Transl Med. 2013;5:186ra66.

3. Botling J, Edlund K, Lohr M, Hellwig B, Holmberg L, Lambe M, et al. Biomarker discovery in non-small cell lung cancer: integrating gene expression profiling, meta-analysis, and tissue microarray validation. Clin Cancer Res. 2013;19:194-204.

4. Lee ES, Son DS, Kim SH, Lee J, Jo J, Han J, et al. Prediction of recurrence-free survival in postoperative non-small cell lung cancer patients by using an integrated model of clinical information and gene expression. Clin Cancer Res. 2008;14:7397-404.

5. Zhu CQ, Ding K, Strumpf D, Weir BA, Meyerson M, Pennell N, et al. Prognostic and predictive gene signature for adjuvant chemotherapy in resected non-small-cell lung cancer. J Clin Oncol. 2010;28:4417-24.

6. Fouret R, Laffaire J, Hofman P, Beau-Faller M, Mazieres J, Validire P, et al. A comparative and integrative approach identifies ATPase family, AAA domain containing 2 as a likely driver of cell proliferation in lung adenocarcinoma. Clin Cancer Res. 2012;18:5606-16.

7. Hou J, Aerts J, den Hamer B, van Ijcken W, den Bakker M, Riegman P, et al. Gene expression-based classification of non-small cell lung carcinomas and survival prediction. PLoS One. 2010;5:e10312.

8. Kuner R, Muley T, Meister M, Ruschhaupt M, Buness A, Xu EC, et al. Global gene expression analysis reveals specific patterns of cell junctions in non-small cell lung cancer subtypes. Lung Cancer. 2009;63:32-8.

9. Lu Y, Lemon W, Liu PY, Yi Y, Morrison C, Yang P, et al. A gene expression signature predicts survival of patients with stage I non-small cell lung cancer. PLoS Med. 2006;3:e467.

10. Okayama H, Kohno T, Ishii Y, Shimada Y, Shiraishi K, Iwakawa R, et al. Identification of genes upregulated in ALK-positive and EGFR/KRAS/ALK-negative lung adenocarcinomas. Cancer Res. 2012;72:100-11.

11. Der SD, Sykes J, Pintilie M, Zhu CQ, Strumpf D, Liu N, et al. Validation of a histology-independent prognostic gene signature for early-stage, non-small-cell lung cancer including stage IA patients. J Thorac Oncol. 2014;9:59-64.

12. Raponi M, Zhang Y, Yu J, Chen G, Lee G, Taylor JM, et al. Gene expression signatures for predicting prognosis of squamous cell and adenocarcinomas of the lung. Cancer Res. 2006;66:7466-72.

13. Bild AH, Yao G, Chang JT, Wang Q, Potti A, Chasse D, et al. Oncogenic pathway signatures in human cancers as a guide to targeted therapies. Nature. 2006;439:353-7.

14. Brambilla C, Laffaire J, Lantuejoul S, Moro-Sibilot D, Mignotte H, Arbib F, et al. Lung squamous cell carcinomas with basaloid histology represent a specific molecular entity. Clin Cancer Res. 2014;20:5777-86.

15. Xie Y, Xiao G, Coombes KR, Behrens C, Solis LM, Raso G, et al. Robust gene expression signature from formalin-fixed paraffin-embedded samples predicts prognosis of non-small-cell lung cancer patients. Clin Cancer Res. 2011;17:5705-14.
